# Effectiveness of a cognitive behavioral therapy-integrated, hospital-based program for prediabetes: a matched cohort study

**DOI:** 10.1038/s41598-024-58739-8

**Published:** 2024-04-05

**Authors:** Chaiwat Washirasaksiri, Withada Pakornnipat, Pinyapat Ariyakunaphan, Chayanis Kositamongkol, Chaiyaporn Polmanee, Lukana Preechasuk, Naris Jaiborisuttigull, Tullaya Sitasuwan, Rungsima Tinmanee, Pornpoj Pramyothin, Weerachai Srivanichakorn

**Affiliations:** 1 Division of Ambulatory Medicine, Department of Medicine, Faculty of Medicine Siriraj Hospital, Mahidol University, 2 Wang Lang Road, Bangkok Noi, Bangkok, 10700 Thailand; 2 Department of Medicine, Faculty of Medicine Siriraj Hospital, Mahidol University, Bangkok, Thailand; 3 Siriraj Diabetes Center of Excellence, Mahidol University, Bangkok, Thailand; 4 Preventive and Health Promotion Nursing Unit, Faculty of Medicine Siriraj Hospital, Mahidol University, Bangkok, Thailand; 5 Division of Nutrition, Department of Medicine, Siriraj Hospital, Mahidol University, Bangkok, Thailand

**Keywords:** Cognitive behavioral therapy, High cardiometabolic risk, Prediabetic state, Preventive program, Thai, Endocrinology, Disease prevention

## Abstract

Intensive lifestyle interventions are effective in preventing T2DM, but evidence is lacking for high cardiometabolic individuals in hospital settings. We evaluated a hospital-based, diabetes prevention program integrating cognitive behavioral therapy (CBT) for individuals with prediabetes. This matched cohort assessed individuals with prediabetes receiving the prevention program, which were matched 1:1 with those receiving standard care. The year-long program included five in-person sessions and several online sessions covering prediabetes self-management, dietary and behavioral interventions. Kaplan–Meier and Cox regression models estimated the 60-month T2DM incidence rate. Of 192 patients, 190 joined the prevention program, while 190 out of 10,260 individuals were in the standard-care group. Both groups had similar baseline characteristics (mean age 58.9 ± 10.2 years, FPG 102.3 ± 8.2 mg/dL, HbA1c 5.9 ± 0.3%, BMI 26.2 kg/m^2^, metabolic syndrome 75%, and ASCVD 6.3%). After 12 months, the intervention group only showed significant decreases in FPG, HbA1c, and triglyceride levels and weight. At 60 months, the T2DM incidence rate was 1.7 (95% CI 0.9–2.8) in the intervention group and 3.5 (2.4–4.9) in the standard-care group. After adjusting for variables, the intervention group had a 0.46 times lower risk of developing diabetes. Therefore, healthcare providers should actively promote CBT-integrated, hospital-based diabetes prevention programs to halve diabetes progression.

## Introduction

Diabetes represents a substantial global public health concern. It has been projected that by 2045, the worldwide diabetic population will exceed 783 million, based on an analysis of year 2021 data from the International Diabetes Federation^1^. In Thailand, data from the Thai National Health Examination Survey indicate a rising prevalence of diabetes and individuals at risk within the Thai population^2,3^. Prediabetes is defined by fasting plasma glucose (FPG) levels ranging from 100 to 125 mg/dL, 2-h oral glucose tolerance test levels between 140 and 199 mg/dL, and/or hemoglobin A1c (HbA1c) levels between 5.7 and 6.4%. Prediabetes, also known as impaired fasting glucose, represents a high-risk condition for the development of type 2 diabetes mellitus (T2DM). Globally, prediabetes is associated with an annual T2DM conversion rate of approximately 5% to 10%^4,5^.

Lifestyle modification programs that encompass dietary, behavioral, and physical-activity interventions have proven effective in preventing and delaying the progression from prediabetes to T2DM. A meta-analysis has indicated that intensive lifestyle modification programs exhibit lower T2DM incidence rates at 3 years compared to control groups, with rates of approximately 14% and 23% in the intensive lifestyle and standard groups, respectively^6^. The trial Diabetes Prevention Program in the United States (US DPP) provided an intensive lifestyle intervention with a 16-session core curriculum to participants with impaired glucose tolerance over a 24-week period. The results showed that goal-oriented interventions involving 7% weight loss and 150 min per week of moderate physical activity reduced the incidence of T2DM by 58%^7–9^. The US Centers for Disease Control and Prevention^10^ expanded the scope of the US DPP through the National DPP, a 12-month program incorporating the successful elements of the trial. These include setting realistic goals for weight loss and physical activity, emphasizing self-efficacy, enhancing problem-solving skills, leveraging social supports, and fostering adaptability to change^11,12^. Despite the proven effectiveness of the US DPP and National DPP in promoting weight loss and preventing diabetes progression, challenges in implementation due to significant demands for manpower, funding, and time have impeded their adoption in many countries, especially those with limited resources. The critical challenge remains to develop scalable interventions that are both efficacious and feasible in real-world, hospital-based settings, particularly for adoption in Asian countries with fewer resources.

Cognitive behavioral therapy (CBT) serves as an essential tool for correcting maladaptive behaviors such as substance abuse and poor dietary and exercise habits. It also provides considerable benefits for mental health disorders, including anxiety and depression. The utility of CBT in facilitating lifestyle modifications, especially in managing and maintaining weight among individuals with obesity and Type 2 diabetes, has been substantiated^13–15^. Research conducted in Thailand demonstrates that the application of CBT can significantly improve glycemic control (HbA1c levels) and alleviate diabetes-related distress. Therefore, it is recommended to incorporate CBT with dietary and physical activity interventions to enhance and widen the adoption of healthy lifestyles in those with prediabetes^16^.

A previous study in Thailand reported that community-based behavior change programs for individuals with impaired glucose tolerance increased knowledge and understanding of self-care and reduced the 2-year incidence of T2DM^17^. Nevertheless, information is scarce regarding effective strategies for managing high cardiometabolic risk individuals with impaired fasting glucose and/or HbA1c-based prediabetes within hospital-based settings. These settings may differ in several aspects, including the risk of T2DM progression, comorbidity prevalence, healthcare circumstances, self-care proficiency, disease management, and medication usage. Consequently, this study was designed to assess the efficacy of a hospital-based diabetes prevention program integrating cognitive behavioral therapy compared to comprehensive standard care for high cardiometabolic risk individuals with prediabetes.

## Results

**Figure 1:**
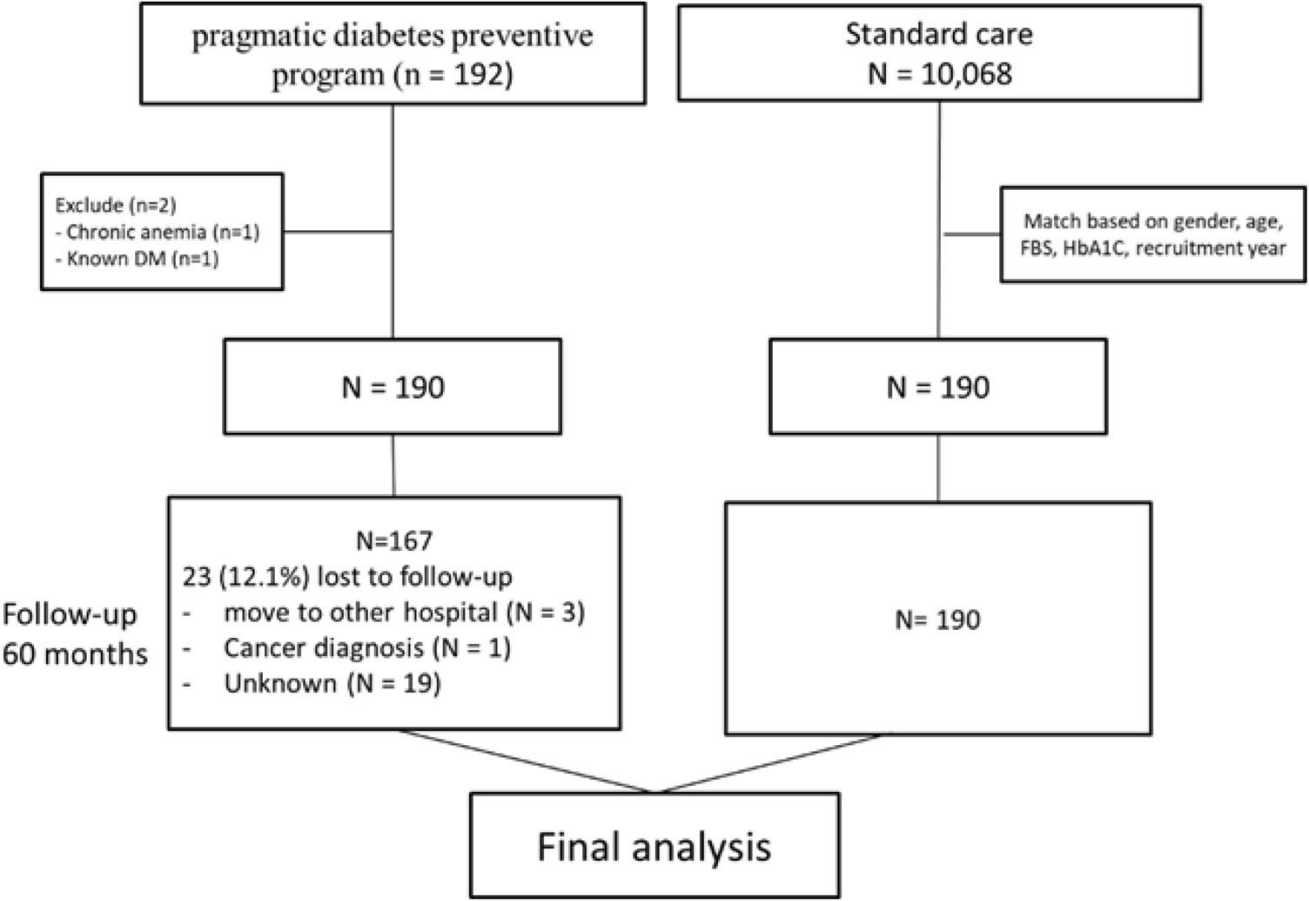
Participant enrollment and follow-up flowchart.

Two patients in the intervention group were excluded due to having chronic anemia or prior knowledge of T2DM before joining the program. One hundred ninety individuals in the intervention group and another 190 in the standard care group met the inclusion and matching criteria, as depicted in Fig. 1. Among the 380 participants, 79.5% were female, 75% had metabolic syndrome, and 6.3% had previously been diagnosed with atherosclerotic cardiovascular disease. The mean age of the participants was 58.9 ± 10.2 years, with a mean HbA1c of 5.9% ± 0.3%, mean FPG of 102.3 ± 8.2 mg/dL, and mean BMI of 26.2 ± 4.7 kg/m^2^. Baseline characteristics were comparable between the two groups, with a higher proportion of statin use and a higher mean diastolic blood pressure in the standard care group (Table 1).

**Table 1:** Baseline demographic and clinical characteristics of the study cohort.

Baseline characteristics	Intervention group (n = 190)	Standard care (n = 190)	*P*
Male, n	39 (20.53)	39 (20.53)	1.0
Age (years)	58.84 (10.23)	58.94 (10.22)	0.9
Weight (kg)	65.40 (14.40)	65.39 (11.05)	0.7
BMI (kg/m^2^) (SD)	26.41 (5.30)	25.93 (4.07)	0.3
SBP (mmHg)	130.93 (16.52)	133.59 (13.40)	0.08
DBP (mmHg)	74.20 (12.21)	78.91 (10.64)	< 0.001
FBS (mg/dL)	102.11 (8.39)	102.52 (7.94)	0.3
HbA1C^19^	5.86 (0.29)	5.86 (0.28)	0.8
Total cholesterol (mg/dL)	199.89 (37.93)	199.40 (40.13)	0.9
LDL-c (mg/dL)	115.39 (34.42)	119.68 (41.00)	0.2
HDL-c (mg/dL)	59.74 (15.57)	57.70 (14.44)	0.2
TG (mg/dL)	125.21 (69.86)	128.56 (67.37)	0.6
AST (U/L)	21.0 (17.0, 26.0)	21.0 (18.0, 26.0)	0.7
ALT (U/L)	19.0 (14.0, 27.5)	19.50 (14.0, 26.0)	0.7
ALP (U/L)	71.63 (16.76)	71.82 (19.32)	0.9
Uric acid (mg/dL)	5.70 (1.35)	5.61 (1.45)	0.5
GFR (mL/min)	84.90 (16.57)	83.38 (18.08)	0.4
BUN (mg/dL)	12.40 (3.12)	13.30 (3.76)	0.3
Cr (mg/dL), mean	0.82 (0.20)	0.83 (0.21)	0.5
MCV (fL), mean	85.86 (8.64)	87.73 (6.47)	0.1
Metabolic syndrome, n	142 (74.4)	134 (74.9)	0.5
Antihypertensive use, n	84 (45.4)	97 (52.4)	0.1
Statin use, n	37 (19.47)	74 (38.94)	< 0.001

Data are presented as the mean ± standard deviation, n [16], or median (25th–75th percentile).

*ALP* alkaline phosphatase, *ALT* alanine aminotransferase, *AST* aspartate aminotransferase, *BMI* body mass index, *BUN* blood urea nitrogen, *Cr* creatinine, *DBP* diastolic blood pressure, *FBS* fasting blood sugar, *GFR* glomerular filtration rate, *HbA1c* hemoglobin A1c, *HDL-c* high-density lipoprotein cholesterol, *LDL-c* low-density lipoprotein cholesterol, *MCV* mean corpuscular volume, *SBP* systolic blood pressure, *SD* standard deviation, *TG* triglycerides.

After 12 months of intervention, participants in the intervention group exhibited significant reductions in body weight, BMI, and levels of fasting glucose, HbA1c, and triglycerides. The median body weight decreased by approximately 0.7 kg in the intervention group, whereas it remained unchanged in the standard care group. Additionally, the intervention group showed mean reductions of 1 mg/dL in FPG and 0.1% in HbA1c, while the standard care group experienced an increase of 2 mg/dL in FPG and no change in HbA1c.

A comparison of the baseline and 60-month data revealed that median diastolic blood pressure increased by 2.0 mmHg in the intervention group but decreased by 2.0 mmHg in the standard care group (*P* < 0.001). Regarding aspartate aminotransferase, a median decrement of 0.5 U/L was observed in the intervention group, while an increment of 2.0 U/L was noted in the standard care group (P 0.02). No significant changes were observed in the median differences of all other parameters (Table 2).

**Table 2:** Comparative median variations in participant characteristics at baseline, 12 months, and 60 months.

Characteristic differences	12-month follow-up Δ (B-12 mo)			60-month follow-up Δ (B-60 mo)		
Median differences	Intervention	Standard care	***P***	Intervention	Standard care	*P*
ΔBMI (kg/m^2^)	0.32 (− 0.23, 0.91)	0.00 (− 0.37, 0.69)	0.005	0.30 (− 0.43, 1.20)	0.04 (− 0.58, 1.18)	0.3
*Δ*BW (kg)	0.70 (− 0.60, 2.40)	0.00 (− 1.00, 1.63)	0.003	0.70 (− 1.05, 3.00)	0.00 (− 1.35, 2.95)	0.3
ΔFBS (mg/dl)	1.00 (− 4.00, 7.00)	− 2.00 (− 7.00, 5.00)	0.01	− 1.00 (− 10.75, 3.75)	− 3.00 (− 9.00, 4.00)	0.5
ΔHbA1C^19^	0.10 (− 0.10, 0.30)	0.00 (− 0.10, 0.20)	0.04	0.00 (− 0.30, 0.20)	− 0.10 (− 0.30, 0.10)	0.4
ΔSBP (mmHg)	2.00 (− 6.25, 12.00)	0.00 (− 9.50, 10.00)	0.3	− 1.00 (− 10.00, 8.75)	− 0.50 (− 11.75, 9.00)	0.2
ΔDBP (mmHg)	− 1.00 (− 7.25, 6.00)	1.00 (− 6.00, 8.00)	0.08	− 2.00 (− 8.00, 4.75)	2.00 (− 4.00,10.00)	0.001
ΔCholesterol (mg/dL)	1.50 (− 15.25, 24.25)	0.00 (− 9.00, 23.50)	0.7	7.00 (− 11.75, 36.00)	14.00 (− 11.75, 40.75)	0.4
ΔLDL-c (mg/dL)	1.40 (− 12.95, 20.70)	1.70 (− 8.30, 18.80)	0.4	7.00 (− 8.60, 31.80)	12.40 (− 5.70, 39.50)	0.1
ΔHDL-c (mg/dL)	− 1.00 (− 7.00, 2.00)	0.00 (− 5.00, 5.00)	0.05	− 3.00 (− 7.00, 5.00)	0.00 (− 5.00, 7.00)	0.2
ΔTG (mg/dL)	7.00 (− 11.75, 34.75)	− 2.00 (− 23.00, 23.00)	0.01	4.00 (− 15.00, 37.00)	0.50 (− 23.25, 27.25)	0.3
ΔCr (mg/dL)	0.01 (− 0.05, 0.06)	0.00 (− 0.04, 0.05)	0.4	0.01 (− 0.06, 0.07)	0.01 (− 0.04, 0.07)	0.9
ΔGFR (mL/min)	− 0.27 (− 4.96, 4.21)	0.56 (− 2.64, − 3.84)	0.2	− 3.03 (− 12.74, 3.94)	2.35 (− 3.09, 6.40)	0.1
ΔAST, (U/L)	0.00 (− 5.75, 5.00)	0.00 (− 4.50, 1.50)	0.05	0.50 (− 5.00, 5.75)	− 2.00 (− 6.00, 2.00)	0.02
ΔALT, (U/L)	0.00 (− 3.00, 4.00)	− 0.50 (− 4.00, 4.00)	0.1	0.00 (− 4.75, 5.75)	− 1.50 (− 7.00, 5.00)	0.2
New statins, n	30 (19.6)	27 (23.3)	0.4	62 (32.6)	63 (33.6)	0.9

*ALT* alanine aminotransferase, *AST* aspartate aminotransferase, *B* baseline, *BMI* body mass index, *BW* body weight, *Cr* creatinine, *dl* deciliter, *DBP* diastolic blood pressure, *FBS* fasting blood sugar, *GFR* glomerular filtration rate, *HbA1c* hemoglobin A1c, *HDL-c* high-density lipoprotein cholesterol, *kg/m*^2^ kilogram per meter squared, *LDL-c* low-density lipoprotein cholesterol, *mg* milligram, *min* minute, *mo* month, *SBP* systolic blood pressure, *TG* triglycerides.

**Figure 2:**
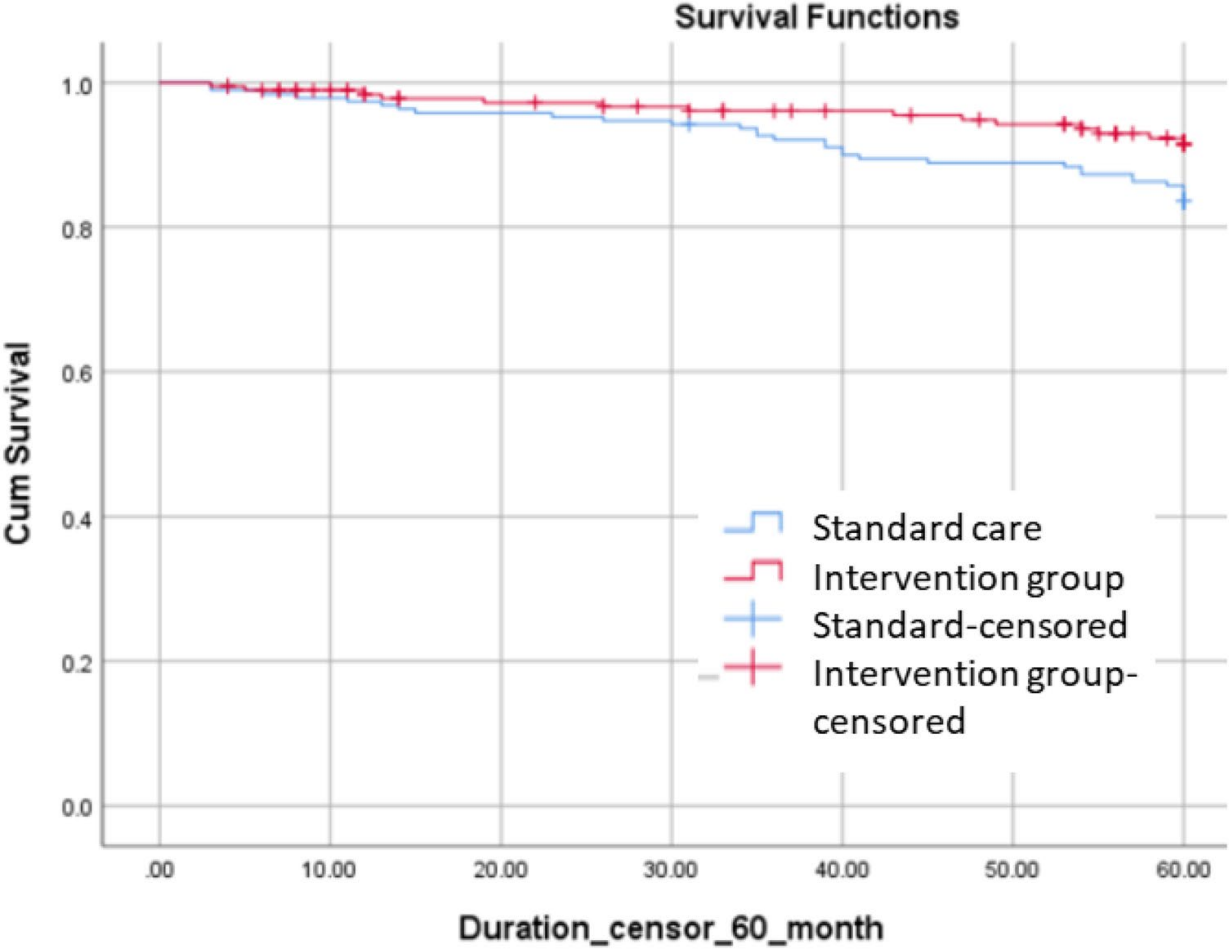
Kaplan–Meier survival curves for the incidence of type 2 diabetes mellitus over a 60-month follow-up period.

Turning to T2DM incidence, 14 participants (8.4%) in the intervention group and 31 (16.3%) in the standard care group developed T2DM within 60 months. The incidence rate (cases per 100 person-years) of diabetes was 1.7 (95% CI 0.9 to 2.8) in the intervention group and 3.5 (95% CI 2.4 to 4.9) in the standard group (Table 3). After adjusting for age, sex, BMI, blood pressure, statin use, FPG, HbA1c, ALT, and HDL, the multivariable-adjusted hazard ratio of incident diabetes in the intervention group compared to standard care was 0.46 (95% CI 0.21 to 0.98, Fig. 2).

**Table 3:** Cumulative incidence rates of type 2 diabetes mellitus during the study period.

	Intervention group (n = 167)	Standard care (n = 190)
T2DM diagnosed (n)	14	31
Incidence proportion (%)	8.4%	16.3%
Total follow-up time (years)	822.1	885.1
Median follow-up duration (years)	5	5
T2DM incidence rate (cases per 100 person-years)	1.7	3.5

*T2DM* type 2 diabetes mellitus.

## Discussion

Numerous studies have highlighted the benefits of diabetes prevention programs for individuals with nondiabetic hyperglycemia during the trial period and during long-term follow-ups ranging from 7 to 20 years^6,9,17–20^. However, the present study stands among the few that have demonstrated the effectiveness of a 1-year intervention program alongside standard care for high cardiometabolic individuals in Thailand with prediabetes. In line with previous research, our study revealed that a 1-year intervention period, compared to standard care alone, significantly reduced body weight, BMI, and glycemia^7,21–23^. The intervention group and the standard care group showed no significant differences in changes in body weight, BMI, glycemia, or dyslipidemia between baseline and the end of the follow-up period. However, our hospital-based diabetes prevention program, which included cognitive behavioral therapy, successfully reduced the incidence of diabetes over the 5-year follow-up.

The extent of weight loss varies among studies, depending on the level of lifestyle intensification and initial body weight^24^. In the current study, at the 1-year follow-up, participants in the intervention group experienced a modest weight reduction of approximately 0.7 kg. However, their weight remained stable over the subsequent 5 years. The weight loss achieved in our study is lower than that achieved in Caucasian populations in a Finnish diabetes prevention study, the US National DPP, and the US DPP^9,12,21^. However, the modest weight loss is consistent with results from prior studies, such as the Da Qing Impaired Glucose Tolerance and Diabetes Study in China and the Diabetes Prevention Program in India^18,19^. Several factors could explain the minimal weight loss. First, the Asian population typically exhibits lower BMI and initial body weight than the Caucasian population. Second, our hospital-based lifestyle program consisted of only five sessions, whereas previous studies offered more intensive programs with approximately 12–16 sessions per year^21,25,26^. Our findings were compatible with a less intensive 1-year intervention program, which was a community-based translation of the Finnish DPS targeting those at high risk for type 2 diabetes in Athens, Greece^27^. The pattern of weight regains observed after the prevention program in our study is also consistent with findings from prior long-term research^20^.

Previous cohort and randomized controlled trials across various ethnicities have reported that the incidence of prediabetes progressing to diabetes in standard control groups varies widely, from 9.3 to 67.7%^9,17,28^. In Asian cohorts, the cumulative incidence was 55.0% over 3 years among Asian Indians^19^. 66.7% over 6 years in a Chinese population^18^, and 16.6% over 2 years within a Thai community-based study^17^, all of which exceed the incidence rates found in our research. These disparities may be attributable to the differing clinical profiles and conventional treatment approaches for at-risk individuals. Unlike our participants, who had impaired fasting glucose and/or HbA1c levels indicating prediabetes, individuals in most previous studies had impaired glucose tolerance. The incidence rates of diabetes observed in our study paralleled those in earlier research focusing on individuals with solely impaired fasting glucose and/or HbA1c levels^4,29^. Additionally, our study group presented with marginally lower FPG and/or HbA1c levels, a lower BMI, a higher mean age, and a more substantial decrease in average lipid levels than those in previous studies^9,17–19^. These clinical features, along with baseline glycemic levels, intensive metabolic management strategies, and the particular phenotype of prediabetes, are likely influencing factors in the rate of diabetes progression observed in our study^30^.

This study showed that a comprehensive hospital-based program providing dietary, physical activity, and psychological support combined with proactive outpatient care promotes weight loss, improves glycemic and lipid levels, and reduces diabetes rates. There were no significant differences in the changes from baseline in body weight, BMI, glycemia, and dyslipidemia between the intervention and the standard care groups. The impact on the 5-year diabetes incidence may be due to a legacy effect from earlier weight loss and metabolic improvements.

Supporting evidence from Korea, India, and Sweden indicates that individuals who initially achieved minimal weight reduction with intensive standard care had a lower incidence of diabetes^19,31,32^. In our study, the prevention program effectively averted the onset of diabetes in over half of the high-risk individuals. The reduction in the risk of progression to diabetes achieved through our hospital-based program was approximately equal to or higher than that reported in previous Thai community-based settings^17^.

Our findings are in close agreement with the seminal DPP study in the United States and the Da Qing study in China. These pivotal investigations demonstrated that intensive lifestyle interventions reduced the incidence of diabetes by 58% over a 2.8-year follow-up period in the United States^7^ and by 42% over a 6-year follow-up period in China^18^. Unfortunately, these comprehensive interventions were resource intensive, demanding substantial human resources, time, and funding; they were also applied to high-risk participants. In contrast, our hospital-based diabetes prevention program supplemented standard care, minimized resource utilization, and was suitable for well-managed individuals at high cardiometabolic risk with prediabetes. The study findings confirm the effectiveness of the hospital-based prevention program in reducing the progression of diabetes, particularly in hospital settings with limited resources.

The efficacy of our intervention is linked to its content and intensity. Our strategies are based on established behavior change models in chronic disease and encompass various approaches to prediabetes self-management and group interventions. These interventions offer social support and are likely to be effective in the Thai context. These nonpharmacologic interventions align with previous recommendations for diabetes prevention^33,34^.

While the decreased diabetes incidence in this study may be partly due to a legacy effect, the median diastolic blood pressure improvement was significantly greater in the standard care group. Further analysis indicated a significant reduction in the mean diastolic blood pressure level from baseline in the standard care group (*P* 0.001) but not in the intervention group (*P* 0.1, data not shown). Several factors may explain the variations in diastolic blood pressure levels. For instance, there was greater use of new antihypertensive medications in the standard care group due to their higher initial diastolic blood pressure. Additionally, other risk factors that increase diastolic blood pressure could not be modified by the hospital-based program, such as smoking habits, weight gain during follow-up, and high salt intake^35^.

Regarding the increase in aspartate aminotransferase liver enzymes in the standard care group at the 60-month follow-up, several factors can influence liver function, including alcohol consumption, herbal use, and other medication intake. We noted a higher prevalence of statin usage in the intervention group than in the standard care group. The intervention group’s weight reduction and improved glycemic and lipid levels may have had a beneficial impact on liver health, particularly in the context of fatty liver disease.

Our analysis presents both strengths and limitations. The study is among the few assessing the impact of a year-long hospital-based intervention alongside standard care over a 5-year span in Thai individuals with high cardiometabolic risk and prediabetes, defined by impaired fasting glucose and HbA1c levels. However, there are constraints to consider, primarily stemming from our retrospective cohort study design. Despite our efforts to match the baseline characteristics of the two groups, this design imposes inherent limitations due to the complexity of the progression from prediabetes to diabetes, influenced by multiple factors. A more comprehensive collection of individual data changes over time, including dietary habits, physical activity levels, family diabetes history, and hypertension treatments, would undoubtedly enhance our study. In light of these limitations, we took measures to minimize potential biases and incomplete data records. In the intervention group, data were systematically recorded and monitored from the outset, ensuring a robust dataset. Additionally, standard control data were systematically paired using baseline characteristics from a large dataset. By implementing these rigorous data collection and matching procedures, we have made every effort to mitigate selection bias and minimize incomplete data records, thus strengthening the overall validity of our study.

Another consideration is that the number of participants in the present study was lower than that in previous diabetes prevention studies. However, it is important to note that the sample size in our study was meticulously calculated based on the estimated number of target individuals needed, ensuring that our study had an adequate number of participants. Despite this, a 12.1% loss to follow-up occurred, which could introduce bias into the results. Nevertheless, it is worth noting that the total duration of follow-up was similar in both groups, and the dropout rate fell within an acceptable range based on previous studies (5%–28%)^28^. Furthermore, the determination of sample size and statistical power was primarily based on differences in diabetes incidence rates noted in earlier research, which implies that our findings related to secondary measures should be approached with caution. Further validation of the intervention’s effectiveness is warranted through randomized controlled trials with more extensive participant samples.

Our findings indicate that a hospital-based diabetes prevention program incorporating cognitive behavioral therapy can significantly reduce the long-term incidence of diabetes in individuals with prediabetes. It is crucial for healthcare professionals to offer such prevention programs to individuals with prediabetes and to promote their participation to forestall or delay the onset of diabetes.

## Materials and methods

### Study design and participants

The data for this analysis were obtained from the medical records of registered participants who visited the outpatient clinic of the Faculty of Medicine Siriraj Hospital between January 2013 and December 2016. This clinic accepts self-referrals as well as referrals from primary and secondary care services. Typically, clinic attendees are individuals with a high risk of diabetes or cardiovascular diseases, or those who already have conditions such as hypertension, obesity, or multiple metabolic risk factors. Standard outpatient care comprises education, appropriate treatments, preventive strategies, and medications as deemed necessary. The inclusion criteria for participants in this study were a baseline HbA1c level less than 6.5% (< 48 mmol/mol) and a baseline FPG level below 7.0 mmol/L. Patients were excluded if they were diagnosed with diabetes prior to the baseline measurements, had thalassemia disease or traits, had chronic anemia, were using any hypoglycemic agents or steroids, or were pregnant.

Since 2013, the Siriraj Diabetes Prevention Program (Si-DPP), a hospital-based program integrating cognitive behavioral therapy, has been in operation. This program offers self-management education, fosters self-care skills, and motivates at-risk individuals. We conducted a retrospective matched cohort study within a single center. The study enrolled 192 participants in the Si-DPP (completing at least three out of five sessions) and 10 068 participants who received standard care. Prior to statistical evaluation, we employed a stratified matching approach based on sex, age, FPG and HbA1c levels, and year of recruitment. Age was divided into six categories (< 18, 18.0–39.9, 40.0–49.9, 50.0–59.9, 60.0–69.9, and ≥ 70.0 years). FPG was grouped into three ranges (< 100, 100–109, and 110–125 mg/dL). HbA1c levels were stratified as < 5.7%, 5.7%–5.9%, and 6.0%–6.4%. Recruitment was stratified by year as 2013, 2014, 2015, and 2016. This resulted in 118 strata for matching. Participants in the intervention group were then paired 1:1 with those in the control group using a computerized random selection process. The baseline clinical characteristics and laboratory parameters of the intervention group participants were collected using standard case records and follow-up forms. Similarly, in the matched standard group, data were collected using standard electronic medical records in the outpatient clinic. The study protocol adhered to the principles of the Declaration of Helsinki and was authorized by the Siriraj Institutional Review Board (approval numbers Si-011/2014 and Si-567/2021).

### Study interventions

The Faculty of Medicine Siriraj Hospital, a tertiary care center in Thailand, developed a hospital-based diabetes prevention program integrating cognitive behavioral therapy. The intervention consisted of a 1-year diabetes prevention program comprising five face-to-face sessions scheduled at the first, second, fifth, eighth, and 12th months. Each session, lasting 3–4 h, included prediabetes self-management education (conducted in group settings with 10–12 individuals as well as individually), hands-on self-practice workshops, and behavioral interventions through self-help groups.

The Si-DPP was developed and implemented by a multidisciplinary team, comprised of internists, psychologists, nurses, dietitians, certified diabetes educators, and sports medicine experts. The team was trained in psychological theoretical frameworks, such as cognitive behavioral theory, plan of attack theory and the theory of planned behavior, to enhance the program’s efficacy. The Si-DPP was characterized by personalized goal setting, risk evaluation, nutritional guidance, and physical activity recommendations, all tailored to the individual health, psychological, and socio-ecological profiles of participants^15^. The Si-DPP emphasized the following aspects:

- Reducing caloric intake and quantities of key dietary components such as sugar, fat, and sodium.
- Providing cooking and shopping tips.
- Promoting at least 150 min of moderate-intensity exercise per week.
- Encouraging self-management skills and self-monitoring for prediabetes and associated comorbidities, including the monitoring of weight, waist circumference, and blood pressure.

CBT techniques aimed at altering cognitive beliefs and behaviors were delivered through “self-help groups” facilitated by trained nurses and healthcare professionals. These sessions, integral to the program’s five core sessions and lasting 40–60 min each, included commitment strategies, customized goal setting, barrier identification, problem-solving enhancements, relapse management, and confidence building for change initiation. The CBT framework was adapted to the Thai cultural context, drawing from prior meta-analyses and research within Thailand^15,16^.

Furthermore, supportive online gamification and motivation sessions were conducted between the five face-to-face sessions via social media applications. These online sessions included challenges related to maintaining a healthy diet, engaging in physical activities, replacing sugar-sweetened beverages with water, sharing photographs of healthy activities, and participating in motivational activity contests.

Following the 1-year program, participants in the intervention group underwent follow-up at least twice a year at the outpatient clinic. These appointments enabled their primary doctors and the multidisciplinary team to review progress and provide appropriate support.

### Measures and outcomes

Participants were assessed at baseline for the presence of atherosclerotic cardiovascular disease and metabolic syndrome. “Atherosclerotic cardiovascular disease” was defined as the presence of coronary syndrome, peripheral artery disease, and/or cerebrovascular disease. “Metabolic syndrome” was defined as having an FPG level ≥ 100 mg/dL or an HbA1c level ≥ 5.7%, along with at least two of the following:

- Body mass index (BMI) ≥ 23 kg/m^2^, as per Asian-specific BMI standards^36,37^.
- Documented hypertension.
- Low levels of high-density lipoprotein cholesterol specific to sex^38,39^.
- Hypertriglyceridemia (fasting triglycerides ≥ 150 mg/dL)^38,39^.
- Documented dyslipidemia or statin use^38,39^.

The primary outcomes of the study were the incidence rates of diabetes and the interval to the development of diabetes following the baseline assessment. Diabetes mellitus was indicated by a documented diagnosis by a healthcare professional, oral hypoglycemic agent use, or repeated FPG measurements ≥ 126 mg/dL and/or repeated HbA1c measurements ≥ 6.5%.

The secondary outcomes included changes in various measures at the 1-year and 5-year follow-ups. These measures included mean body weight, BMI, FPG, blood pressure, creatinine, glomerular filtration rate, liver function tests, uric acid, total cholesterol, low-density lipoprotein cholesterol, high-density lipoprotein cholesterol, and triglycerides.

### Statistical analysis and sample size calculation

The sample size for each group was determined based on the diabetes incidence rates from a previous study. In the intervention group, the rate of diabetes was 46.0%, whereas in the standard care group, it was 67.7%^18^ Based on a type I error of 0.05 and a power of 90%, the sample size for each group was calculated using a two-independent proportion formula, taking into account a 20% dropout rate. As a result, the number of participants per group was approximately 190 participants.

Continuous variables with a normal distribution are presented as the means ± standard deviations, while those with a skewed distribution are summarized as the medians with interquartile ranges. Categorical variables are expressed as numbers and percentages. Baseline group comparisons were conducted using independent *t* tests for normally distributed variables and Mann–Whitney U tests for skewed distribution variables. The chi-square test was employed to compare categorical variables.

Changes in weight, BMI, blood pressure, creatinine, glomerular filtration rate, liver function tests, uric acid, and lipid parameters were calculated between baseline and 1 year and within 60 months for each group. Diabetes incidence rates per 100 person-years in the intervention and standard groups were computed and estimated using Kaplan–Meier analysis. Cox regression models were employed to explore the hazard ratios for T2DM incidence in each group. Adjustments were made for age, sex, BMI, blood pressure, statin use, baseline FPG, HbA1c, ALT, and HDL. Statistical analyses were performed using IBM SPSS Statistics (version 28, IBM Corp, Armonk, NY, USA).

### Informed consent

Informed consent was secured from all participants in the study.

## Data Availability

Data from this study are available upon request from the corresponding author. The data are not publicly available due to privacy and ethical reasons.
